# Transcriptional regulatory functions of nuclear long noncoding RNAs

**DOI:** 10.1016/j.tig.2014.06.001

**Published:** 2014-08

**Authors:** Keith W. Vance, Chris P. Ponting

**Affiliations:** MRC Functional Genomics Unit, Department of Physiology, Anatomy and Genetics, University of Oxford, South Parks Road, Oxford, OX1 3PT, UK

**Keywords:** long noncoding RNA, transcription, chromatin conformation, RNA–protein interactions

## Abstract

Several nuclear localised intergenic long noncoding RNAs (lncRNAs) have been ascribed regulatory roles in transcriptional control and their number is growing rapidly. Initially, these transcripts were shown to function locally, near their sites of synthesis, by regulating the expression of neighbouring genes. More recently, lncRNAs have been demonstrated to interact with chromatin at several thousand different locations across multiple chromosomes and to modulate large-scale gene expression programs. Although the molecular mechanisms involved in targeting lncRNAs to distal binding sites remain poorly understood, the spatial organisation of the genome may have a role in specifying lncRNA function. Recent advances indicate that intergenic lncRNAs may exert more widespread effects on gene regulation than previously anticipated.

## Highlights

• Nuclear localised lncRNAs regulate the expression of both local and distal genes.
• lncRNAs can function locally to regulate enhancer–promoter interactions.
• lncRNAs can interact with chromatin at many different locations genome wide.
• RNA–protein–DNA and RNA–DNA interactions guide lncRNAs to their target sites.

## Emerging roles for nuclear lncRNAs

The mammalian genome contains large numbers of noncoding RNA (ncRNA) loci that interdigitate between, within, and among protein-coding genes on either strand. To date, more than 10 000 mammalian intergenic lncRNAs [>200 nucleotides (nt)] (see Glossary) have been catalogued; the majority of these are expressed at lower levels compared with protein-coding transcripts, and are more tissue specific [1–3]. A small number of intergenic lncRNAs have been implicated in a variety of biological processes [4]. The functions, if any, of the remaining transcripts remain unknown and, in contrast to protein-coding sequence, cannot yet be predicted from sequence alone [5].

**Figure 1:**
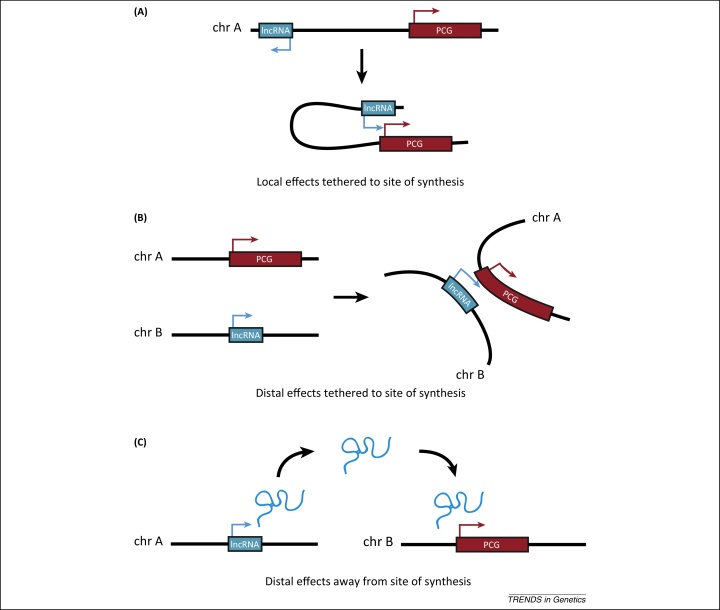
Local and distal modes of long noncoding RNA (lncRNA)-mediated transcriptional regulation. **(A)** DNA looping interactions bring a lncRNA locus into close physical proximity with a genomically adjacent protein coding gene (PCG). Such lncRNAs function close to their sites of synthesis to regulate the expression of nearby genes on the same chromosome. **(B)** Chromatin conformation changes bring two distantly located loci into close spatial proximity. lncRNAs in this category function close to their site of synthesis, but their genomic PCG targets are located on different or homologous chromosomes (chr). **(C)** lncRNAs translocate from their sites of synthesis to regulate transcription of distantly located target genes on the same or different chromosomes.

Some intergenic lncRNAs function as transcriptional regulators that can act locally, near their sites of synthesis, to regulate the expression of nearby genes, or distally to regulate gene expression across multiple chromosomes (Figure 1). Here, we draw upon recent studies to review the functions of nuclear localised intergenic lncRNAs in regulating gene transcription and chromatin organisation, their local and distal modes of action, their mechanisms of genomic targeting, and the nature of their interactions with chromatin.

## lncRNAs function at their sites of synthesis to regulate local gene expression

Intergenic lncRNAs have been divided, on the basis of chromatin marks at their promoters, into two broad categories: those emanating from enhancer regions or those transcribed from promoter-like lncRNA loci [6]. Most, if not all, transcriptional enhancer elements are transcribed to produce often exosome sensitive, unspliced transcripts termed ‘enhancer RNAs’ (eRNAs). The level of these transcripts tends to correlate positively with expression levels of neighbouring protein coding genes [7,8]. A subset of enhancers also appears to be associated with polyadenylated, more stable, and often spliced lncRNAs variously called elncRNAs, 1d-eRNAs, or ncRNA-activating lncRNAs (ncRNA-a) [6,9–11]. All of these transcripts are likely generated bidirectionally with RNAs transcribed from either or both strands being rapidly degraded, as seen for unstable antisense promoter upstream transcripts (PROMPTS) [12] and for intragenic enhancer produced transcripts [13]. Thus, further experiments will be needed to determine the relative proportions and functions of enhancer-associated lncRNA loci that are uni- or bi-directional, capped, and polyadenylated or unpolyadenylated, and multi- or mono-exonic.

It is currently unknown whether eRNAs or elncRNAs are commonly simply a by-product of or an actual cause of enhancer action on neighbouring protein-coding genes. However, a small but growing number of eRNAs and elncRNAs have been shown to function at their site of synthesis in a RNA-dependent manner to regulate positively the expression of neighbouring protein coding genes on the same chromosome [14–18]. In one study, multiple 17B-oestradiol (E2)-induced eRNA transcripts were found to interact with cohesin *in vitro* and to induce looping interactions between their enhancer elements and the promoters of nearby target genes [15]. In another, two elncRNAs (ncRNA-a3 and ncRNA-a7) bound to components of the Mediator complex and also promoted enhancer–promoter looping interactions to regulate local gene expression [19]. In a third study, upon depletion of an eRNA transcribed from the *MyoD1* core enhancer region, both *MyoD1* chromatin accessibility and RNA polymerase II (PolII) occupancy were reduced and *MyoD1* expression was decreased [17]. Therefore, enhancer-associated transcripts can modulate enhancer activity by altering local chromatin accessibility and/or structure. Nevertheless, other studies showed that eRNAs generated from p53-bound enhancers acted on pre-existing chromatin conformations to increase enhancer activity by unknown mechanisms [16] and that inhibition of eRNA production at estrogen receptor (ER) bound enhancers, for example by blocking transcriptional elongation, had no effect on chromatin looping yet still inhibited target gene activation [18]. Thus, enhancer-associated lncRNAs may have multiple RNA-dependent mechanisms of transcriptional control.

lncRNA loci have also been ascribed RNA-independent functions in gene activation, for example that arise from transcription through loci affecting local chromatin accessibility, as described during *fbp1*+ gene activation in yeast [20]. In another example, the activity of the human growth hormone (*hGH*) HS1 enhancer was shown to be stimulated by lncRNA transcription that initiates immediately downstream of HS1 and is noncontiguous with the *hGH* target promoter [21]. To investigate the molecular mechanism of this stimulation, a transcriptional terminator was inserted into the locus, which led to reduced lncRNA transcription, and to a concomitant decrease in *hGH* expression. When the sequence of this lncRNA was replaced by that of an unrelated bacteriophage RNA, the enhancing effect of the natural transcript was recapitulated. Taken together, these studies show that enhancer-associated lncRNAs can also act locally, near their site of synthesis, using either RNA-dependent or -independent mechanisms to increase the transcriptional activity of chromosomally proximal protein coding genes.

Local changes in gene expression are presumed to be mediated by *cis*-acting lncRNA modes of action on the same chromosome, and in an allele-specific manner. However, *trans*-acting lncRNA mechanisms could also operate to control nearby gene expression. The lncRNA *Jpx*, for example, is transcribed from the active X chromosome and can upregulate expression of the adjacent *Xist* gene on the other future inactive X allele in *trans* during the process of X chromosome inactivation [22]. *Evf-2*, in addition, modulates the activity of an enhancer element present within its locus by binding distal-less homeobox 2 (DLX2) and methyl CpG binding protein 2 (MECP2) and inhibiting DNA methylation of this enhancer to control expression of the genomically adjacent *Dlx6* gene [23,24]. These effects occur in *trans* because *Evf-2* and DLX2 cooperate to increase the activity of the *Dlx6* enhancer when they are co-expressed from transiently transfected plasmids in a reporter assay, and *Evf-2* inhibits DNA methylation when expressed ectopically from a transgene in a mouse model. In general, further mechanistic studies will be needed to assess the relative contributions of *cis*- and *trans*-acting lncRNA mechanisms controlling local gene expression.

## lncRNAS with distal regulatory functions

Many experiments thus far have focussed on the possible mechanisms by which an enhancer-associated lncRNA or transcription of its locus regulates the expression level of an adjacent protein-coding gene. However, long-range intra-chromosomal interactions between eRNA expressing loci and distantly located loci have also been documented [15,16]. The *TFF1* and *NRIP1* eRNA containing loci, located 27 Mb apart on chromosome 21, are brought into close spatial proximity by long-range DNA looping interactions. This looping is induced by E2 and appears to be dependent on the *NRIP1* eRNA. Therefore, a subset of eRNAs may have so far uncharacterised distal regulatory roles.

What has been studied less often is whether lncRNA transcripts can function in *trans* at distal genomic locations. To address this and other issues, several techniques have been recently developed [25–28] to map the occupancy of lncRNAs genome-wide (Box 1). Although these approaches are providing insights into the direct or indirect binding of RNAs to genomic locations, it is important also to understand their limitations. One of these is that false positive inferences can arise from the direct DNA binding of the antisense oligonucleotides used to capture the RNA and also from the cross-linking of spatially adjacent genomic regions in the nucleus. Interpretation of results is further complicated by the observation that the binding of transcription factors to DNA is often insufficient to alter transcription [29,30]. Thus, we expect that a large number of lncRNA binding events are also inconsequential. For example, one eRNA, transcribed from an E2-regulated *FoxC1* enhancer, has been shown to occupy 15 binding locations on multiple chromosomes, all well away from its endogenous locus; however, none of these were located within regulatory regions of E2-responsive genes and, thus, probably represent nonfunctional genomic interactions [15]. Therefore, to prioritise binding events that are functional, it is necessary to identify genes that are both directly bound and regulated by lncRNA transcripts, for example by intersecting lncRNA genomic binding profiles with lncRNA-induced gene expression changes.

Determination of the genomic binding profiles for several other types of lncRNA shows that a single lncRNA transcript can interact with multiple binding sites on different chromosomes away from its site of transcription. *Hotair*, a lncRNA transcribed from the homeobox (*Hox) C* locus, was shown initially to function in *trans* to repress the transcription of genes in the *HoxD* gene cluster on another chromosome [31]. Subsequently, it was found to associate with approximately 800 binding locations of up to 1 kb in length across multiple chromosomes. These focal binding sites are reported to be embedded within larger polycomb domains and are enriched within genes that become derepressed upon *Hotair* depletion [25]. Another lncRNA, prostate-specific transcript 1 (non-protein coding) (*PCGEM1*), which binds to the androgen receptor (AR), associates with 2142 binding locations on the genome, the majority (approximately 70%) of which correspond to AR-bound H3K4me1-modified enhancers. *PCGEM1* association with AR-bound enhancers appears to increase AR-mediated gene activation without affecting AR levels [32].

*Paupar* is an intergenic lncRNA that interacts with chromatin at over 2800 sites located on multiple chromosomes and controls large-scale gene expression programs in a transcript-dependent manner [33]. It is transcribed from a conserved enhancer upstream of the paired box 6 (*Pax6*) gene and its depletion significantly alters the expression of *Pax6* and 942 other genes distributed across the genome. *Paupar* binding sites, defined by CHART-Seq (Box 1), overlap functional elements, such as DNase I hypersensitive sites (HSs), and are enriched at gene promoters. Control CHART-Seq experiments using lacZ probes showed that binding to such sites by RNAs can be nonspecific. Consequently, candidate functional *Paupar* binding sites were predicted to be only those within the regulatory regions of genes that were differentially expressed upon *Paupar* depletion. *Paupar* was then shown using reporter assays to modulate the transcriptional activity, in *trans* and in a dose-dependent manner, of three out of five such candidate regions tested. These experiments demonstrate that a lncRNA can have dual functions both locally, to regulate the expression of its neighbouring protein coding gene, and distally at regulatory elements genome-wide. In this case, the distal functions of *Paupar* rely, in part, on it being guided to its genomic binding sites by formation of a complex with PAX6, a DNA-binding protein.

The *Firre* lncRNA also appears, from its genome-wide binding profile, to act locally as well as distally. It occupies a large 5-Mb domain surrounding its site of synthesis on the X chromosome and interacts with five additional domains on four different autosomes [34]. Only one of these binding events was shown to alter the expression of a gene within the bound region. The ncRNA *Ctbp1-as* has also been shown to function both locally, to repress *Ctbp1* expression through a sense-antisense mediated mechanism, and distally to increase AR transcriptional activity in prostate cancer cells [35].

**Figure 2:**
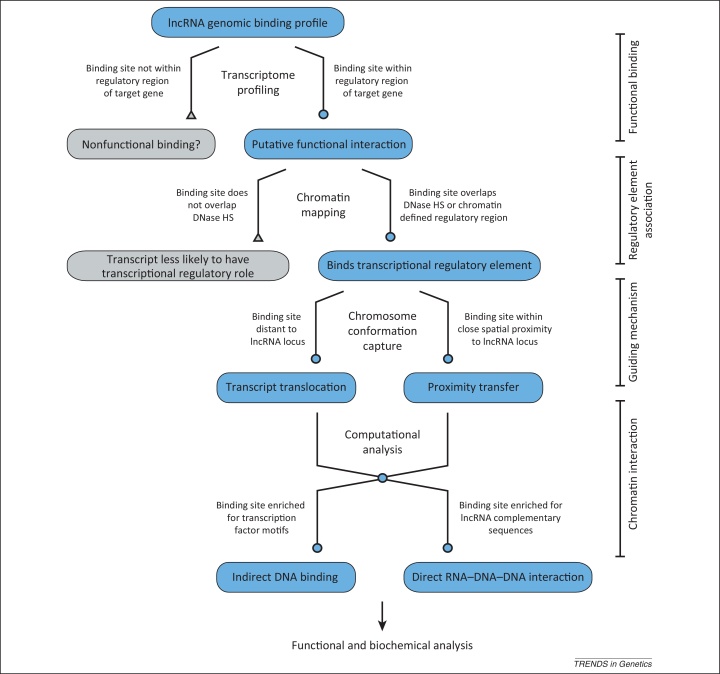
Workflow diagram detailing a combined experimental and computational pipeline for investigating *trans*-acting long noncoding RNA (lncRNA) transcriptional regulatory functions. Likely functional lncRNA genomic binding sites are identified within the regulatory regions of target genes that are differentially expressed upon lncRNA depletion or overexpression. Gene Ontology analyses of lncRNA bound and regulated genes provide clues regarding lncRNA function. Binding sites that associate with transcriptional regulatory regions are further selected for based on DNase I hypersensitive site mapping and chromatin status. Putative functional binding locations are integrated with chromosome conformation capture-based experiments to provide insights into the mechanism of genomic targeting. Computational sequence analyses generate predictions regarding how lncRNAs interact with DNA. These criteria are used to inform the design of reporter assays and biochemical experiments aimed at understanding *trans*-acting lncRNA function and mode of action at candidate loci.

Although such studies are as yet limited in number, they suggest that the ability of lncRNAs to function both locally, as well as distally, to regulate large-scale gene expression programs may be more widespread than originally anticipated. As genome-wide binding profiles for more lncRNAs are mapped and their direct transcriptional targets are identified, there will be increasing opportunities to elucidate their presumed heterogeneous molecular mechanisms (Figure 2).

## lncRNA genome targeting

The mechanisms by which lncRNAs target specific genomic sequences are not understood. It is easy to envisage that lncRNA transcripts could participate in regulating local gene expression by accumulating to comparatively high concentrations at their sites of synthesis. However, it is more difficult to explain how lowly expressed, and often unstable, nuclear lncRNAs act by binding many different chromosomal regions that lie distant to their site of transcription. Recent reports suggest that the 3D conformation of the genome guides lncRNAs to distal binding sites. This process of ‘proximity transfer’ was first proposed for *Xist* on the basis of its transferral from its site of synthesis to distal, yet spatially close, binding sites along the X chromosome; however, confirmation of this model will require data at higher resolution than the 1-Mbp intervals used in the initial study [26].

The mechanism of proximity transfer is further supported by observations concerning the *Hottip* lncRNA locus. Chromosomal looping interactions were found that brought this locus into close spatial proximity to its target genes in the *HOXA* cluster [36]. Furthermore, transcription was activated when the *Hottip* transcript was recruited to its target promoters in reporter assays, whereas induction of *Hottip* expression from an ectopic site had no effect [36]. The spatial organisation of the genome might also permit lncRNAs to span multiple binding locations across different chromosomes, including their sites of synthesis. Consistent with this, the binding domains of *Firre* on different chromosomes appear to be located in close spatial proximity within the nucleus [34].

Several nuclear lncRNAs are able to regulate transcription when expressed in *trans* from ectopic loci [22,24,33,37]. This suggests an alternative model in which lncRNAs are translocated from their site of synthesis as components of ribonucleoprotein complexes to bind specifically and regulate the expression of distantly located target genes. One such lncRNA could be *NeST*, which is involved in controlling the immune response to microbial infection. *NeST* can activate interferon gamma (*Ifn)g* transcription in *trans*, both when expressed from a transgene and also from its genomic locus, by interacting with WD repeat domain 5 (WDR5) and by altering *Ifng* histone H3K4 tri-methylation [37]. Additionally, and in contrast to the proximity transfer model, *Xist* might be a diffusible factor; when *Xist* is expressed from a transgene in female mouse embryonic fibroblasts, it diffused from the ectopic site of synthesis and acted on the endogenous *Xist* locus in *trans* [38]. Similarly, *Evf-2*, when expressed from a transiently transfected plasmid, cooperated with the DLX2 protein to activate the *Dlx-5/6* enhancer in a luciferase reporter in *trans* [24], and depletion of the endogenous *Paupar* transcript modulated, in a dose-dependent manner, the transcriptional activity of a number of its genomic binding sites when inserted into transiently transfected reporters [33].

Therefore, coordination among sites of lncRNA synthesis, the spatial organisation of the genome in the nucleus, and specific lncRNA interactions with transcription and chromatin regulatory proteins are all likely to have roles in facilitating binding of lncRNA transcripts to their genomic targets (Figure 2).

## lncRNA–chromatin interactions

**Figure 3:**
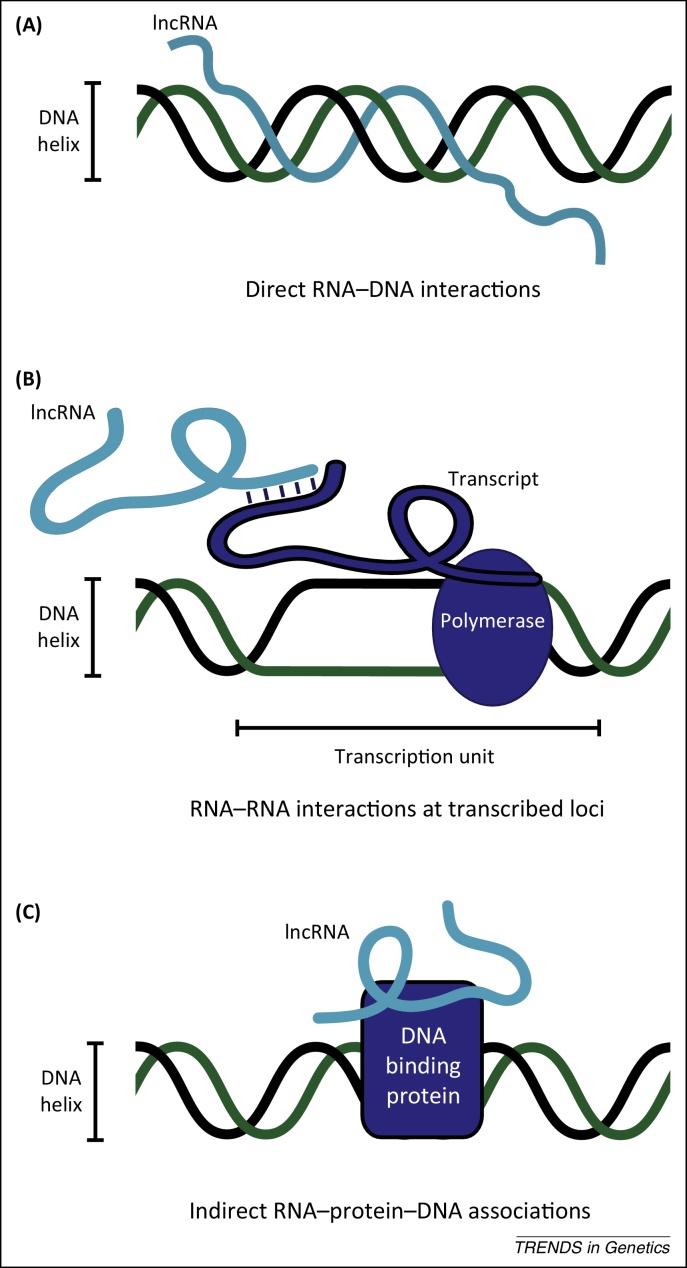
Different modes of long noncoding RNA (lncRNA)–chromatin association. **(A)** Single-stranded lncRNAs directly interact with complementary double-stranded DNA target sequences through hydrogen bonding to form a RNA-DNA-DNA triplex structure. lncRNAs are predicted to bind in the major groove of the DNA through either Hoogsteen or reverse Hoogsteen base pairing. **(B)** lncRNAs base pair with RNA sequences at transcribed loci. This may involve Watson–Crick base pairing (G–C, A–U) between complementary nucleotides as well as non-Watson–Crick base pairing (G–U, A–A) which does not require exact sequence complementarity. **(C)** Indirect recruitment of lncRNAs to the genome through RNA–protein–DNA interactions. This includes lncRNA associations with sequence specific DNA binding transcription factors, non-DNA binding transcriptional cofactors and histone proteins. Post-translational histone modifications, such as acetylation, methylation, and ubiquitination, may influence lncRNA–histone binding.

The genomic associations observed between lncRNAs and chromatin could be accomplished through direct base pairing between RNA and DNA sequences [39] (Figure 3A). This is exemplified by promoter associated RNA (pRNA), a low-abundance RNA transcribed from upstream of the pre-rRNA transcription start site that can interact with complementary sequences within the rDNA promoter forming a RNA–DNA–DNA triplex, possibly through Hoogsteen base-pairing [40]. Furthermore, because RNA base pairs with itself, RNA–RNA interactions between complementary sequences at transcribed loci could also guide lncRNAs to their genomic targets (Figure 3B). It remains unclear how widespread direct RNA–DNA or RNA–RNA targeting may be.

lncRNAs that associate with sequence-specific DNA binding transcription factors could be targeted to the genome indirectly through RNA–protein–DNA interactions (Figure 3C). YY1, for example, is a zinc finger-containing transcription factor that may recruit *Xist* to chromatin by binding DNA and *Xist* RNA through different sequence domains [38]. Other candidates for RNA–protein DNA-binding complexes include *Gas5* and glucocorticoid receptor [41], *Panda* and nuclear transcription factor Y (NFYA) [42], *Lethe* and nuclear factor kB (NF-kB) [43], *Jpx* and CCCTC-binding factor (CTCF) [44], *Paupar* and PAX6 [33], *Rmst* and SRY (sex determining region Y)-box (SOX2) [45], and *Prncr1* and AR [32]. Each of the *Gas5*, *Panda*, *Lethe*, and *Jpx* lncRNAs appears to inhibit DNA binding of their associated transcription factors at several target sites, whereas knockdown of *Paupar* and *Prncr1* levels had no effect on PAX6 or AR occupancy where tested. By contrast, *Rmst* appears to be required for the correct association of SOX2 with promoter regions of neurogenic target genes [45]. Therefore, lncRNAs can actively modulate the DNA binding activity of their associated transcription factors as well as acting as non-DNA binding cofactors, as has been described for *Six3OS* and *SRA* [46,47], whose precise regulatory roles need to be investigated.

Chromatin modification and structure may also modulate how lncRNAs are recruited to the genome. *Xist* appears to target active chromatin because domains that are initially occupied by *Xist* are unusually enriched in actively transcribed genes and open chromatin [48], whereas AR-associated lncRNAs *Pcgem1* and *Prncr1* preferentially interact with enhancer-associated histone modifications *in vitro* [32]. Computational approaches are beginning to predict how lncRNAs interact with chromatin or DNA: firstly, by suggesting the candidature of lncRNA-associated transcription factors from enrichments of their binding motifs; secondly by proposing the involvement of the lncRNA in transcriptional enhancement or repression from enrichments of relevant chromatin marks; and thirdly by identifying near complementary DNA sequence within lncRNA-associated regions that might indicate direct RNA–DNA–DNA triplex formation [25,33,49,50].

## Mechanisms of action

Several lncRNAs associate with chromatin-modifying complexes and transcriptional regulatory proteins in the nucleus. High throughput RNA-immunoprecipitation (RNA-IP) experiments have indicated that individual chromatin-modifying complexes, such as polycomb repressive complex 2 (PRC2) and mixed-lineage leukemia (MLL), interact with thousands of RNA transcripts, including lncRNAs [51,52]. However, substantial numbers of transcripts are known to bind nonspecifically and reproducibly with various RNA-binding proteins in RNA-IP based assays [53]. Furthermore, purified PRC2 complex, for example, binds RNA nonspecifically *in vitro* in a size-dependent manner [54]. Thus, studies will need to distinguish specific from nonspecific RNA–protein interactions.

lncRNAs that bind proteins specifically might act as guides to target chromatin-modifying complexes to the genome. The lncRNA *Mistral*, for example, which is transcribed from the intergenic region between the *Hoxa6* and *Hoxa7* genes, forms a RNA–DNA hybrid structure at its site of synthesis which recruits MLL1 complex proteins [55]. By contrast, the lateral mesoderm-specific lncRNA *Fendrr* can associate with PRC2 and regulate *Pitx2* expression in *trans*. *Fendrr* is predicted to interact with a short stretch of complementary sequence, of fewer than 40 nt, in the *Pitx2* promoter, which can form a RNA–DNA–DNA triplex *in vitro.* Given that *Pitx2* promoter PRC2 occupancy and histone H3K27 tri-methylation are decreased in *Fendrr* knockout embryos, *Fendrr* may have a role in recruiting PRC2 to the *Pitx2* promoter [56]. Thus, such nuclear lncRNAs have the potential to target chromatin remodelling complexes either to their sites of synthesis or to distally located loci in *trans*.

Several recent studies suggest that lncRNAs modulate the structure and function of their associated protein complexes. The *Drosophila roX2* lncRNA appears to function as a critical complex subunit that is necessary for the correct assembly of a functional male-specific lethal (MSL) dosage compensation complex. Its stem loop-containing structured domains bind the MLE RNA helicase and MSL2 ubiquitin ligase components of the MSL dosage compensation complex in a sequential manner [57,58]. Interaction of *roX2* with the MLE RNA helicase results in an ATP-dependent conformational change in a *roX2* stem-loop structure and a subsequent increase in its association with MSL2. Other lncRNAs may also promote the ordered recruitment of functional ribonucleoprotein complexes. For example, *Pcgem1* and *Prncr1* bind AR sequentially, thereby stimulating both ligand-dependent and ligand-independent AR controlled gene expression programs [15]. *Prncr1* first associates with DOT1-like, histone H3 methyltransferase (DOT1L) and binds to acetylated enhancer-bound AR causing DOT1L to methylate the AR. AR methylation subsequently induces recruitment of *Pcgem1*, in complex with pygopus homolog 2 (PYGO2), which because of its H3K4me3-binding ability, may stimulate DNA looping interactions between AR-bound transcriptional enhancers and target promoters.

These studies raise the possibility that a single lncRNA molecule contains multiple structural motifs, upon which multiple different proteins bind, which enhance the efficiency of genomic targeting and transcriptional regulation [59]. *Hotair*, for example, interacts with PRC2 through its 5′ end, whereas its 3′ region associates with (co)repressor for element-1-silencing transcription factor (CoREST) *in vitro* [60]. A scaffolding role for lncRNAs may also translocate gene loci between different nuclear compartments to allow transcriptional activation or repression in response to various stimuli. This is exemplified by the differential interactions of taurine upregulated 1 (*Tug1*) and metastasis associated lung adenocarcinoma transcript 1(*Malat1*) with methylated and unmethylated Pc2 protein, respectively, and the subsequent relocation of growth control genes from *Tug1*-containing polycomb bodies, where they are repressed, to interchromatin granules for assembly of activator complexes, where they are activated [61].

## Concluding remarks

Roles for lncRNAs as regulators of chromatin organisation and gene expression were initially described for *H19* and *Xist* lncRNAs in genomic imprinting and X chromosome inactivation, respectively. It has since become apparent that the genome encodes large numbers of nuclear localised intergenic transcripts. The number of these transcripts that derive from serendipitous transcription or that fail to have functions (as opposed to mere effects [62]) remains unknown. Evolutionary evidence for selected effect functionality [62] of lncRNAs, in general, is meagre [3,63] and the proportion of lncRNA sequence that is under purifying selection appears to be small, approximately 5% [64]. Therefore, further detailed studies on a larger sample of lncRNAs are needed to estimate the proportion of lncRNAs that are functional, as well as to define their structure–function relations, and to better understand the mechanisms of these transcripts in regulating genome organisation and gene transcription. We also await the results of lncRNA loss-of-function studies in animal model systems that discriminate lncRNA from DNA sequence-mediated effects that might identify nuclear lncRNAs that are essential for embryonic development and adult tissue homeostasis *in vivo*.

## Box 1 Mapping genomic binding sites of lncRNAs

Four techniques have been developed recently that map the interaction of RNA with chromatin: chromatin oligoaffinity precipitation (ChOP); chromatin isolation by RNA purification (ChIRP); capture hybridisation analysis of RNA targets (CHART); and RNA antisense purification (RAP) [25–28,50]. Each uses biotinylated antisense oligonucleotides to capture RNA from cross-linked chromatin extracts, in combination with quantitative (q)PCR and/or high-throughput sequencing, to identify their associated DNA binding sites. Although these techniques are based on an analogous concept, they differ in their chromatin cross-linking, shearing, and hybridisation conditions, the size and number of oligonucleotide probes used to capture target RNAs, and the method of elution of the associated DNA fragments. Whereas the ChOP method has only been used to examine RNA occupancy at discrete loci [28,65], ChIRP, CHART, and RAP have been applied to map RNA chromatin occupancy genome-wide.

A pool of approximately 24 short 20-nt probes are used in ChIRP to enrich for RNA targets from nonreversible glutaraldehyde cross-linked extract [25], whereas in RAP a large pool (1054 probes for the 17-kb *Xist* transcript) of long 120-nt capture oligonucleotides are used to pull down target transcripts from a combination of glutarate and formaldehyde cross-linked cells [26]. The use of capture oligonucleotides that span the whole length of the target molecule in both ChIRP and RAP has been reported to improve the effectiveness of this approach to uniformly capture long RNAs [25]. Moreover, when RAP was used to purify the highly abundant *Xist* transcript from female lung fibroblasts, approximately 70% of the total RNA-Seq reads in the pull down corresponded to *Xist* transcript, representing a massive enrichment [26]. CHART, by comparison, uses RNaseH sensitivity mapping to design a small number of 18–28-nt antisense oligonucleotides against regions of the target RNA that are accessible for hybridisation [48,50]. These are then used to capture RNA targets from formaldehyde cross-linked chromatin. In the CHART protocol, hybridisation is performed at room temperature and RNaseH is used to elute RNA–chromatin complexes that specifically interact with antisense capture DNA oligonucleotides, which is effective in reducing the number of false positive interactions that are generated due to direct DNA binding of antisense oligonucleotides.

The size, abundance, and subcellular localisation of the target RNA are likely to influence the efficacy of these approaches and, therefore, the optimal method should be determined for each transcript.

## Glossary

*Cis*-acting lncRNA: a lncRNA that functions close to its site of synthesis to regulate the expression of nearby genes on the same chromosome in an allele-specific manner.
Enhancer-associated lncRNA: a lncRNA whose genomic locus is marked by high levels of histone H3 lysine 4 mono- compared to tri-methylation.
Intergenic lncRNA: a lncRNA whose genomic locus does not overlap transcribed protein coding gene sequence.
lncRNA: an RNA molecule, greater than 200 nt in length, which is not predicted to encode protein.
Promoter-associated lncRNA: a lncRNA whose genomic locus is marked by high levels of histone H3 lysine 4 tri-methylation relative to monomethylation.
Proximity transfer: translocation of an RNA molecule from its site of transcription to distal binding sites, located in close spatial proximity.
*Trans*-acting lncRNA: a lncRNA that regulates the expression of genes on a different chromosome and/or on the homologous chromosome from where it is transcribed.
